# Biomaterials for Improving Skin Penetration in Treatment of Skin Cancer

**DOI:** 10.3390/jfb17010039

**Published:** 2026-01-15

**Authors:** Davide Secci, Andrew Urquhart, Vasileios Bekiaris, Katrine Qvortrup

**Affiliations:** 1 Department of Heath Technology, Technical University of Denmark, DK-2800 Lyngby, Denmark; davsec@dtu.dk (D.S.); anur@dtu.dk (A.U.); vasbek@dtu.dk (V.B.); 2 Department of Chemistry, Technical University of Denmark, DK-2800 Lyngby, Denmark

**Keywords:** biomaterials, drug delivery, skin barrier, skin cancer, topical administration

## Abstract

Skin cancer is among the most common malignancies worldwide, posing a significant societal burden due to its increasing incidence and its limited responsiveness to conventional topical therapies. Treatment is challenged by the presence of the skin barrier which restricts drug penetration. This review discusses the structural and physiological challenges of topical delivery and summarizes efforts to develop functional biomaterials to enhance skin drug penetration. Important aspects to consider when developing formulation strategies, such as drug properties and release mechanisms, are discussed alongside current limitations and future perspectives.

## 1. Introduction

Globally, skin cancer remains one of the most widespread forms of malignancy, with increasing incidence rates due to factors such as aging population, increased UV exposure, and improved surveillance [1,2].

Cutaneous melanoma, accounting for approximately 5% of all skin cancer diagnoses, is responsible for the majority of skin cancer-related deaths due to its high metastatic potential and aggressive clinical development [3,4].

Skin cancers are broadly classified into non-melanoma and melanoma according to their cellular origin. Non-melanoma skin cancers primarily include Basal cell carcinoma (BCC) and Squamous cell carcinoma (SCC), which arise from epidermal keratinocytes and account for approximately 95% of all skin cancers. In contrast, melanoma deriving from melanocytes is less frequent, but is associated with high mortality [2,4]. Other rare skin tumors include Merkel cell carcinoma, Kaposi’s sarcoma, and cutaneous lymphomas such as Mycosis fungoides. Clinically and diagnostically, skin cancer lesions are often organized into epidermal, melanocytic, and other malignant or benign categories, a structure that reflects their histopathology and guides clinical management [5,6].

These tumor types differ in their responsiveness to topical therapeutic approaches. While non-melanoma skin cancers typically remain confined to superficial epidermal or upper dermal layers, invasive melanoma is characterized by rapid vertical growth and early breach of the basement membrane, which substantially limits the effectiveness of localized topical drug delivery.

Conventional treatments such as surgical excision, chemotherapy, radiotherapy, and immunotherapy remain the first-in-line strategies for skin cancer management. Surgical resection is generally effective for early-stage diseases, whereas systemic therapies, including targeted agents and immune checkpoint inhibitors, are used in advanced melanoma [7]. However, their success is often constrained by systemic toxicity, the emergence of drug resistance, and incomplete tumor eradication, particularly in metastatic settings [8,9].

Topical drug delivery is a promising alternative, providing the opportunity to deliver therapeutic agents directly to the affected site while minimizing systemic exposure [10]. However, the skin’s protective barrier, particularly the stratum corneum, poses a major obstacle by preventing drug penetration and retention, challenging the ability to achieve therapeutically effective concentrations in deeper skin layers and tumor tissue [11].

To address these challenges, biomaterial-based delivery systems have been developed to enhance local drug deposition, prolong release, and improve bioavailability [12]. Recent advances in nanotechnology and biomaterials engineering have enabled the design of carriers with tunable size, surface chemistry, and responsiveness, capable of overcoming physiological barriers and enhancing drug transport through the skin [13]. Nanocarriers such as liposomes, polymeric nanoparticles, and solid lipid nanoparticles can improve solubility, stability, and targeted delivery of anticancer agents [12,13]. In parallel, microneedle arrays and hydrogel-based depots provide minimally invasive platforms that achieve sustained, localized release and controlled dosing, with potential benefits in skin cancer therapy [12,13,14].

In this review, the term “nanocarrier” refers to drug delivery systems with characteristic sizes typically ranging from approximately 20 to 300 nm, although exact dimensions may vary depending on formulation class and material composition.

This review summarizes current progress in biomaterial-assisted drug delivery for skin cancer, with a focus on their mechanisms of action, formulation strategies, and translational potential.

## 2. Skin Physiology and Permeability

The skin is the body’s largest organ and functions as a complex, multilayered interface between the internal environment and external stimuli [15]. Structurally, it is organized into three principal layers: the epidermis, dermis, and hypodermis. The epidermis, a stratified squamous epithelium, is avascular and the main responsible for the skin barrier protective functions [16].

The epidermis is further divided into five strata that reflect progressive keratinocyte differentiation: the stratum basale, composed of proliferative keratinocytes and melanocytes anchored to the basement membrane; the stratum spinosum, providing structural support through desmosomal connections; the stratum granulosum, characterized by keratohyalin granules and the establishment of tight junctions; the stratum lucidum, an extra thin layer present only in thick, glabrous skin such as palms and soles; and the stratum corneum, consisting of anucleated, flattened corneocytes embedded in a structured lipid matrix [17,18].

Beneath the epidermis, the basement membrane forms a specialized interface that separates it from the dermis, providing structural support and regulation of molecular exchange between the two layers [19].

The dermis provides structural integrity and elasticity through an extracellular matrix rich in collagen and elastin fibers. It also houses an extensive vascular and lymphatic network, nerve endings, and a variety of skin appendages, including sebaceous glands associated with hair follicles and eccrine and apocrine sweat glands. Eccrine glands are widely distributed across the body and open directly to the skin surface, whereas apocrine glands are localized to specific regions and empty into hair follicles [20,21].

**Figure 1:**
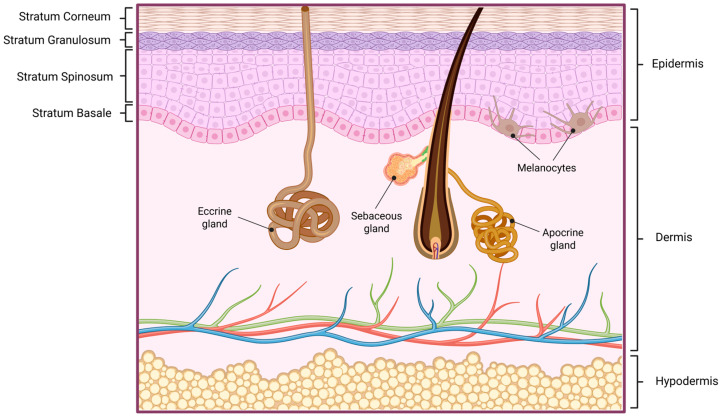
Schematic representation of human skin and its layers. *Created in BioRender. Secci, D. (2026) https://BioRender.com/b9m8yv4 (Accessed on 1 December 2025)*.

The hypodermis, the deepest layer, consists largely of adipose tissue that serves as an energy reservoir, thermal insulator, and mechanical cushion, while anchoring the skin to underlying structures [22]. Figure 1 illustrates the human skin layers.

The skin contains a diverse array of specialized cell populations that sustain its structural and immunological functions. Keratinocytes are metabolically active and proliferative in the lower epidermal layers, particularly in the stratum basale and spinosum. As they migrate upward through the stratum granulosum, they progressively lose their nuclei and organelles, accumulate keratin, and undergo cornification, a specialized form of programmed cell death. By the time they reach the stratum corneum, they have transformed into flattened, anucleated corneocytes, dead cells that form a cohesive and mechanically resilient layer [23]. This structure is stabilized by cornified envelopes and tightly packed lipid lamellae composed predominantly of ceramides, cholesterol, and free fatty acids, creating a dense and selective barrier. Although non-viable, corneocytes serve physiological functions by limiting transepidermal water loss, impeding the entry of xenobiotics and pathogens, and providing a durable mechanical shield [24].

Melanocytes, located in the basal layer of the epidermis, synthesize melanin within specialized organelles called melanosomes, which are transferred via dendritic processes to surrounding keratinocytes, forming a supranuclear ‘melanin cap’ that protects nuclear DNA from UV radiation. However, melanocytes can proliferate abnormally, leading to infiltration of surrounding tissues and deeper skin layers, developing metastatic dissemination [4,25].

In the epidermis, Langerhans cells and specialized T cells together with keratinocytes, which are armed with pattern recognition receptors, form the first line of immunological defense. In the dermis, fibroblasts produce collagen and elastin, providing mechanical support, while dermal immune cells, including macrophages, dendritic cells, and various T cell subsets, orchestrate inflammatory and regulatory responses [26]. Together, these cellular elements enable the skin to act as both a physical barrier and an immunologically active organ.

**Figure 2:**
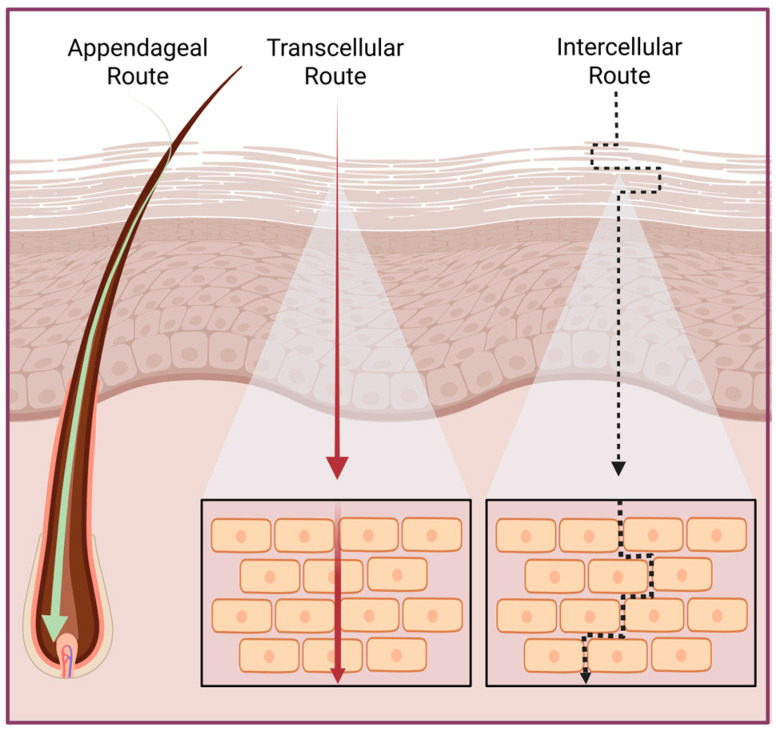
“Brick and mortar” representation and primary topical penetration routes. *Created in BioRender. Secci, D. (2026) https://BioRender.com/g0cj3ph (Accessed on 1 December 2025)*.

The barrier function of the stratum corneum is disproportionately strong relative to its thin structure of approximately 10–20 µm [18]. As shown in Figure 2, this layer is organized into a densely packed “brick-and-mortar” structure of corneocytes embedded within a lipid matrix [27]. The combination of tightly packed corneocytes, the highly organized lipid matrix, a slightly acidic surface pH, tight junctions in the underlying granular layer, and active enzymatic processes create an exceptionally impermeable structure [11].

Molecules can penetrate the skin via three primary routes. The intercellular route, which involves diffusion through the tortuous lipid domains between corneocytes, is the dominant pathway for most small and moderate lipophilic molecules. The transcellular route requires sequential partitioning into and across corneocytes and the intercellular lipid matrix and is less commonly exploited due to the heterogeneous nature of the intracellular environment [28]. The appendageal route, through hair follicles, sebaceous glands, and sweat ducts, bypasses much of the stratum corneum, enabling enhanced delivery of macromolecules, particulate carriers, and advanced transdermal systems such as microneedles and nanocarriers. Although the contribution of the appendageal route under passive conditions is typically limited, it plays an increasingly important role in modern drug delivery strategies designed to overcome the skin barrier [29,30].

## 3. Challenges in Skin Drug Delivery

While the skin barrier is essential for maintaining physiological homeostasis, it also represents a major obstacle to the penetration of therapeutic agents, making transdermal and topical drug delivery inherently challenging.

In topical drug delivery, skin penetration refers to the entry of a compound into superficial skin layers, whereas skin permeation describes its transport across the skin into deeper tissues or systemic circulation. Nanocarriers do not necessarily cross the skin barrier as intact structures, but may instead enhance local drug accumulation or release their payload within specific skin compartments, depending on their physicochemical properties. Understanding the multilayered organization of skin is therefore fundamental to the rational design of advanced drug delivery systems.

The stratum corneum severely limits the permeation of most molecules, especially if they possess high hydrophilicity and molecular weight. As a result, only small, moderately lipophilic molecules with favorable physicochemical properties can passively diffuse across intact skin [31].

In addition to this physical barrier, biological factors further complicate topical drug delivery. Drugs applied to the skin are subject to enzymatic degradation, pH fluctuations, and environmental instability, all of which may prevent drugs in reaching the target site. Cutaneous enzymes, such as peptidases and esterases, are widely expressed in skin layers, where they can significantly alter the fate of topical therapeutics [32]. Furthermore, the skin exhibits a well-defined pH gradient, with acidic conditions in the stratum corneum and a more neutral environment in deeper layers, showing diverse enzymatic activity and barrier homeostasis. All these factors combined can influence both drug stability and bioavailability, thereby reducing effective permeation and altering drug retention [33].

**Figure 3:**
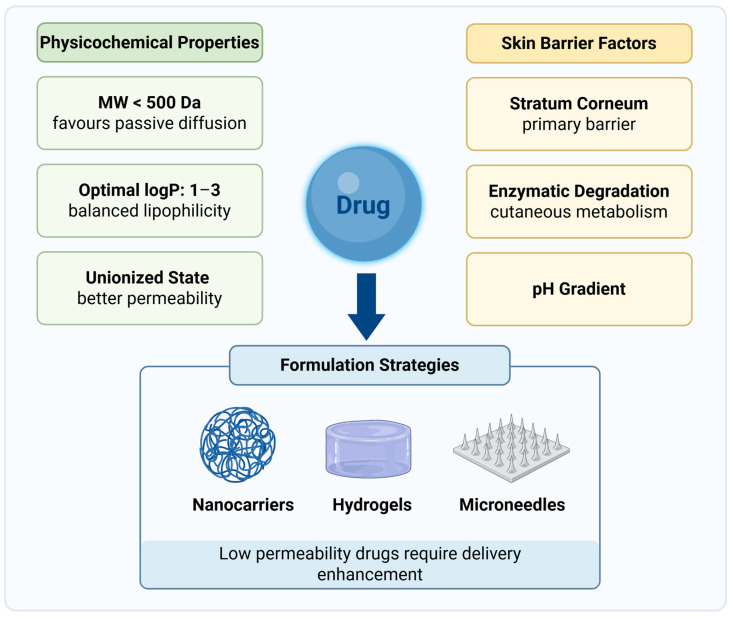
Physicochemical and biological determinants of topical drug delivery. *Created in BioRender. Secci, D. (2026) https://BioRender.com/atf58bo (Accessed on 1 December 2025)*.

The intrinsic physicochemical properties of the drug are critical determinants of its ability to permeate the skin. Hydrophilic molecules, compounds with a molecular weight above approximately 500 Da [34], or those with low octanol/water partition coefficient (logP) values often show poor penetration. Passive skin permeation is generally optimal for compounds with a logP between 1 and 3, as this range ensures adequate partitioning into the lipid-rich stratum corneum while still allowing diffusion toward deeper layers [35]. Compounds with lower logP values are too hydrophilic to cross the barrier, whereas excessively lipophilic molecules tend to remain trapped in the stratum corneum [36]. Figure 3 summarizes the drug physicochemical properties and biological factors that influence skin permeability and proposes formulation strategies.

Beyond basic descriptors such as molecular weight and log P, more advanced parameters like the skin permeability coefficient (Kp) provide a quantitative measure of a compound’s ability to cross the skin barrier. Early models, such as the Potts and Guy equation, demonstrated that skin permeability can be reasonably predicted using lipophilicity (logP) and size (molecular weight), laying the foundation for permeability modeling [37]. Over time, however, this approach has been expanded by incorporating additional molecular descriptors to improve predictive accuracy and account for more structurally diverse compounds [38]. As a result, log Kp remains a central parameter in dermal and transdermal drug delivery research, serving as a practical bridge between physicochemical properties and expected skin permeation behavior.

The drug ionization state plays an additional critical role: only the unionized fraction can efficiently diffuse across the lipophilic barrier, while ionized species are generally excluded or have severely limited permeability. This behavior is strongly influenced by the surrounding pH, making the formulation environment a crucial design parameter [39]. The distribution coefficient (LogD) reflects a compound’s effective lipophilicity at a specific pH, accounting for both ionized and unionized species. Unlike Log P, which represents only the neutral form, LogD provides a more accurate predictor of skin permeability for ionizable molecules [40,41].

When developing skin cancer therapies, these limitations are particularly important to consider. Many anticancer agents have a low permeability through the skin barrier. This often leads to insufficient accumulation at the tumor site and increases the risk of systemic exposure due to absorption through hair follicles or damaged areas. To address these challenges, recent research has focused on developing nanocarriers, microneedles, and other permeation-enhancing strategies to improve targeted delivery and accumulation in tumor tissues [42].

## 4. Biomaterials for Skin Cancer Drug Delivery

The integration of functional biomaterials into topical and transdermal drug delivery platforms has opened new opportunities for localized cancer therapy. In skin cancer, where the target tissue is directly accessible, biomaterial-based systems provide an opportunity to achieve site-specific drug deposition while minimizing systemic exposure [13,43].

Conventional topical formulations have shown limited efficacy due to the strong barrier function of the stratum corneum, rapid enzymatic degradation, and poor penetration [11]. To overcome these obstacles, a broad range of engineered nanocarrier systems have been developed to enhance skin permeation, improve drug retention, and optimize local bioavailability [44].

These systems employ different mechanisms of interaction with the skin barrier, such as enhancing stratum corneum permeability, promoting hydration, sustaining drug release, enhancing follicular targeting, or bypassing the outermost layers altogether [16]. Additional strategies include loosening the lipid matrix, improving drug partitioning, and responding to physiological stimuli within the skin microenvironment. These versatile mechanisms can be exploited in the context of skin cancer, where localized and controlled delivery plays a vital role in improving therapeutic outcomes [45,46].

This section provides an overview of the principal formulation strategies currently explored for topical and transdermal delivery, encompassing nanoparticle-based systems (I), lipid-based carriers (II), hydrogel matrices (III), and mechanical or physical enhancement approaches (IV). For each class, we discuss their underlying principles, mechanisms of interaction with the skin barrier, therapeutic advantages, and translational considerations in the context of skin cancer.

### 4.1. Polymeric Nanoparticles

Polymeric nanoparticles are nanoscale drug delivery systems built from biocompatible and biodegradable polymers that can encapsulate, adsorb, or conjugate therapeutic agents. Their versatility arises from the controlled functionalization of the polymeric materials, which allows tuning of nanoparticles physicochemical properties, drug release kinetics, and interaction with biological barriers [47]. These systems are particularly suited for topical and transdermal delivery because they can prolong drug retention, protect sensitive compounds from enzymatic degradation, and modulate their penetration through the skin barrier [48].

A wide range of natural and synthetic polymers have been used to design polymeric nanoparticles. Natural polymers such as chitosan, alginate, gelatin, and hyaluronic acid offer inherent biocompatibility and bioadhesion, while synthetic polymers, including poly lactic-co-glycolic acid (PLGA), poly ε-caprolactone (PCL), poly lactic acid (PLA), and polyethylene glycol (PEG) provide better control over degradation rates, drug loading, and release profiles [49].

Structurally, polymeric nanoparticles can be broadly divided into two classical categories: nanospheres and nanocapsules. Nanospheres are solid, matrix-type systems in which the drug is homogeneously dispersed or adsorbed on the surface of the polymer. Their simple architecture allows for stable formulations and controlled drug release through diffusion or erosion of the matrix. Nanocapsules possess a core–shell structure, with the active compound confined within an inner cavity surrounded by a polymeric membrane. This configuration is particularly advantageous for protecting labile compounds and improving the delivery of hydrophobic drugs [50,51].

In addition to these traditional architectures, more specialized polymeric nanostructures have been developed to enhance targeting efficiency and stability. Polymeric micelles, formed by the self-assembly of amphiphilic block copolymers (e.g., PEG–PLA, PEG–PCL), possess a hydrophobic core that solubilizes poorly water-soluble drugs and a hydrophilic corona that improves stability and diffusion through the skin barrier [49,52].

Dendrimers are highly branched, monodisperse macromolecules (e.g., PAMAM, PPI characterized by a central core and multiple terminal functional groups. This dual functionality introduces the possibility to load both hydrophilic and hydrophobic drugs simultaneously, giving formulation structures with precise molecular architecture, and surface functionalization for enhanced penetration and targeting [52,53].

**Figure 4:**
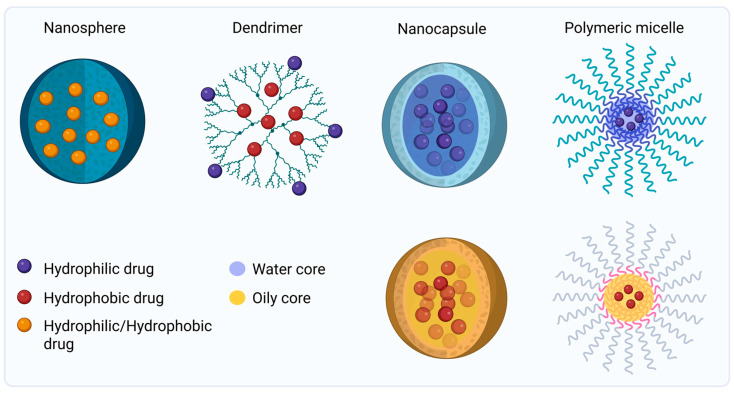
Graphical overview of polymeric nanoparticles delivery systems. *Created in BioRender. Secci, D. (2026) https://BioRender.com/d6u7dee (Accessed on 1 December 2025)*.

Together, these polymeric nanocarriers offer a versatile platform for controlled drug delivery to improve therapeutic efficacy in dermatological and oncological applications [48,50]. Figure 4 illustrates the different types of polymeric nanoparticles delivery systems.

### 4.2. Lipid-Based Nanocarriers

Lipid-based nanocarriers are nanoscale drug delivery systems composed of physiological or synthetic lipids designed to encapsulate, protect, and transport therapeutic molecules across biological barriers. The use of lipids as biomaterials ensures an intrinsic affinity for the stratum corneum lipid matrix, while their excellent biocompatibility and ability to modulate skin permeability make them particularly well suited for topical and transdermal applications. These systems can incorporate both hydrophilic and lipophilic drugs, offering formulation flexibility and the potential to improve local drug bioavailability [54,55].

In the context of skin cancer, lipid-based carriers have gained increasing attention for their capacity to localize therapy at the tumor site, enhance drug penetration, and protect labile anticancer agents from premature degradation [56]. By mimicking the skin’s natural lipid composition, these carriers can achieve efficient permeation and prolonged retention to improve therapeutic outcomes while reducing adverse effects associated with systemic treatments [57].

In this section, lipid-based nanocarriers will be broadly classified into three main categories: (1) nanoemulsions, consisting of dispersed oil and water phases stabilized by surfactants; (2) solid lipid nanoparticles (SLNs) and nanostructured lipid carriers (NLCs), which use solid or mixed solid–liquid lipid matrices for controlled delivery; and (3) vesicular systems, based on phospholipid bilayers capable of encapsulating both hydrophilic and lipophilic molecules.

#### 4.2.1. Nanoemulsions

**Figure 5:**
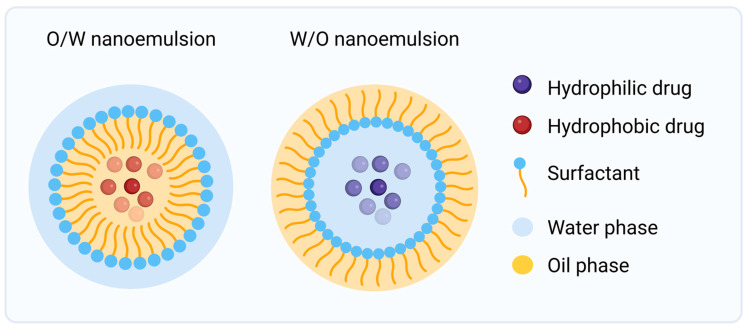
Graphical overview of nanoemulsions drug delivery systems. *Created in BioRender. Secci, D. (2026) https://BioRender.com/nl79lay (Accessed on 1 December 2025)*.

Nanoemulsions are biphasic colloidal dispersions in which one immiscible liquid phase, either oil or water, is dispersed within the other as nanometric droplets, typically ranging from 20 to 200 nm in diameter. As shown in Figure 5, depending on their composition, they can be formulated as oil in water (O/W) or water in oil (W/O) systems [58,59].

They are composed of three fundamental elements: an oil phase, an aqueous phase, and a surfactant system. The oil phase consists of liquid lipids, typically biocompatible triglycerides or fatty acid esters such as medium-chain triglycerides, isopropyl myristate, or natural oils (soybean, olive, sesame, or jojoba) [60]. These act as solvents for hydrophobic drugs and promote biomimetic interactions with the lipid matrix of the skin. The aqueous phase can be supplemented by hydrophilic APIs or co-excipients, buffers, or humectants to maintain osmotic balance, improve hydration, or support drug solubilization depending on the application. The surfactant system is composed of amphiphilic molecules that orient themselves at the interface between the two phases, with their hydrophilic head groups facing the aqueous phase and their hydrophobic tails aligned toward the oil phase. They are broadly classified as non-ionic (tween series and PEG derivatives), anionic (sodium lauryl sulfate), cationic (CTAB and benzalkonium chloride) or zwitterionic (lecithin). When required, a co-surfactant such as a short-chain alcohol or glycol may be incorporated to further enhance droplet formation and improve overall stability [12,61].

These constituents are chosen not only for their stabilizing efficiency but also for their dermal safety and physiological compatibility, in line with biomaterial design principles emphasizing minimal irritation and biodegradability.

Although historically distinguished from nanoemulsions, microemulsions are also nanometric in size, and the distinction between the two lies primarily in their thermodynamic stability and surfactant content. Microemulsions are thermodynamically stable systems that form spontaneously and remain indefinitely dispersed due to their very low interfacial tension but require relatively high surfactant concentrations. In contrast, nanoemulsions are kinetically stable but thermodynamically unstable, needing mechanical energy such as high-pressure homogenization or ultrasonication for droplet formation. Moreover, nanoemulsions achieve nanometric droplet sizes with lower surfactant levels, resulting in improved skin tolerance and broader formulation flexibility [62,63].

Upon application to the skin, nanoemulsion droplets establish close contact with the stratum corneum and enhance percutaneous transport through complementary mechanisms. Their large surface area facilitates intimate contact and increases the concentration gradient across the barrier; surfactants and oil components fluidize the ordered lipid matrix of the stratum corneum to reduce diffusional resistance; and the small droplet size allows penetration through the appendageal route, creating a drug reservoir for sustained release. These synergistic mechanisms result in improved permeation and local retention without the need for additional chemical penetration enhancers, making nanoemulsions suitable for both superficial and transdermal drug delivery [16,47].

In skin cancer treatment, nanoemulsions have been extensively explored as carriers for lipophilic anticancer agents [64]. Their lipidic architecture enhances penetration through hyperkeratotic tumor tissue and supports selective accumulation within malignant epidermal layers, while limiting systemic absorption and toxicity [13,44]. By mimicking the lipid composition of the skin barrier, nanoemulsions exemplify how material design can directly translate into enhanced local bioavailability and safer topical management of skin malignancies.

#### 4.2.2. Solid Lipid Nanoparticles (SLNs) and Nanostructured Lipid Carriers (NLCs)

SLNs are nanoscale carriers composed of biocompatible lipids that remain in a solid state at both physiological and ambient temperatures. These systems are dispersed in an aqueous phase and stabilized by surfactants, forming a rigid lipid core capable of entrapping active molecules within its crystalline network [65,66].

Lipids used for the preparation of SLNs usually have melting points above 40 °C. The solid matrix is generally composed of biocompatible triglycerides such as tristearin or tripalmitin, long-chain fatty acids including stearic acid and palmitic acid or natural and synthetic waxes such as acetyl palmitate, carnauba wax or beeswax [67,68].

The use of solid lipids instead of liquid oils was originally designed to enhance the physicochemical stability of emulsions, reduce drug leakage, and protect the API from oxidation or thermal degradation. Depending on the polarity and solubility of the drug, the active ingredient can be molecularly dispersed, solubilized in the imperfect lipid crystal lattice, or localized at the particle surface [69]. Surfactants such as polysorbates, poloxamers or phospholipids are used to stabilize colloidal dispersion and prevent aggregation through steric or electrostatic repulsion [70].

When applied to the skin, SLNs adhere closely to the stratum corneum, forming an occlusive lipid film that reduces transepidermal water loss, increases local hydration, and enhances drug penetration. The lipid core also promotes partial fusion or lipid exchange with the intercellular matrix, improving drug partitioning into the epidermis and favoring reservoir formation within deeper layers [71]. These processes contribute to higher local drug concentrations, sustained release, and reduced systemic diffusion, properties that are particularly advantageous for the topical treatment of skin cancers, where localized retention and minimal off-target exposure are essential for safety and efficacy.

Despite their biocompatibility and excellent stability, conventional SLNs are limited by a relatively low drug-loading capacity. The highly ordered crystalline arrangement of solid lipids may expel incorporated molecules during storage or polymorphic transitions, leading to drug leakage and decreased encapsulation efficiency [72]. To address these drawbacks, a second generation of lipid nanoparticles known as NLCs was developed. NLCs are based on mixtures of solid and liquid lipids, typically in ratios of 70:30 or 80:20, creating a less ordered matrix with structural imperfections that accommodate higher amounts of drug [68,73]. The introduction of liquid lipids such as oleic acid, isopropyl myristate or medium-chain triglycerides disrupts the crystalline packing, minimizing drug expulsion and allowing greater flexibility for modulation of the release profile.

From a mechanistic perspective, both SLNs and NLCs interact dynamically with the stratum corneum. Their occlusive behavior enhances fluidization with the lipid matrix, while their nanoparticle size enables tight contact and possible follicular deposition. Moreover, the lipid components act as biocompatible penetration enhancers, transiently disrupting the lamellar structure of the skin barrier without causing irritation or structural damage. The resulting increase in skin permeability is often synergistic with the controlled release characteristics of these formulations, providing sustained therapeutic levels within the epidermal tumor microenvironment [68,71].

Several studies have highlighted the potential of SLNs and NLCs for topical chemotherapy in skin cancer. Lipid carriers loaded with anticancer agents show improved dermal accumulation and cytotoxicity against melanoma and non-melanoma cell lines, with reduced systemic toxicity compared to conventional formulations. Their biocompatible composition further supports chronic administration for localized treatment or chemoprevention of precancerous lesions [74,75].

**Figure 6:**
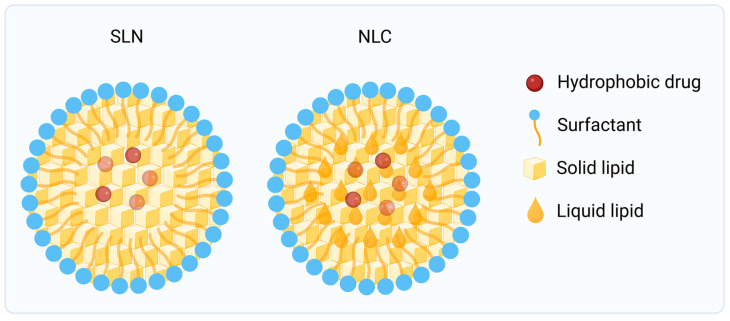
SLNs and NLCs structures. *Created in BioRender. Secci, D. (2026) https://BioRender.com/lk3sveb (Accessed on 1 December 2025)*.

SLNs and NLCs represent versatile lipidic nanoplatforms combining the advantages of traditional emulsions with the stability of solid/mixed matrices. Through rational selection of lipid components and surfactants, these systems can be tailored for specific physicochemical properties, targeting profiles, and release kinetics. In skin cancer therapy, they offer a promising route for site-specific drug delivery, prolonged therapeutic action, and reduced systemic burden. Figure 6 illustrates SLNs and NLCs structures.

#### 4.2.3. Vesicular Systems

Vesicular systems are phospholipid-based nanocarriers composed of one or more concentric bilayers enclosing an aqueous core, forming spherical self-assembled structures that closely mimic biological membranes [76]. Their amphiphilic organization enables dual drug loading, with hydrophilic molecules encapsulated within the aqueous compartment and lipophilic compounds partitioned into the lipid bilayer [77]. Due to their high versatility, vesicular systems such as liposomes, niosomes, ethosomes, and transfersomes have become central in topical and transdermal drug-delivery research [78].

Drug incorporation into these systems can occur either passively or through active (remote) loading. In the passive approach, the drug is entrapped during vesicle formation and distributes between the aqueous and lipid domains according to its solubility and ionization state [79]. Although simple, this method often yields limited encapsulation efficiency and poor retention, particularly for hydrophilic or weakly basic drugs that tend to remain in the external aqueous phase. In contrast, remote loading exploits a pre-established transmembrane pH gradient between the vesicle interior and the external medium, commonly generated using ammonium sulfate or similar buffer systems [80]. The drugs in their neutral form diffuse through the lipid bilayer, where they become ionized and trapped within the vesicle. This mechanism markedly increases loading efficiency and ensures more stable drug retention, making it especially advantageous for hydrophilic anticancer compounds.

The interaction of vesicular carriers with the stratum corneum involves fusion and intercalation with skin lipids, hydration of the corneocyte layer, and temporary disruption of lipid packing, mechanisms that collectively enhance permeation and localized drug deposition [81,82]. Overall, vesicular nanocarriers offer notable advantages including biomimetic composition, high biocompatibility, and formulation versatility. However, their clinical application is still limited by physicochemical instability, high production cost, scale-up challenges, and batch-to-batch variability, which require careful optimization of lipid composition and manufacturing parameters [79].

Depending on their structural organization and physicochemical properties, vesicular systems can be broadly divided into conventional and deformable vesicles. Conventional vesicles, such as liposomes and niosomes, are characterized by relatively rigid bilayers composed of phospholipids or non-ionic surfactants stabilized with cholesterol [83]. These carriers primarily enhance drug permeation through adsorption and fusion with the stratum corneum lipids, followed by gradual diffusion of the drug into deeper skin layers. Their biomimetic composition allows strong affinity with epidermal lipids and provides a protective environment for both hydrophilic and lipophilic molecules, contributing to improved stability and localized retention at the administration site [77,82,84].

**Figure 7:**
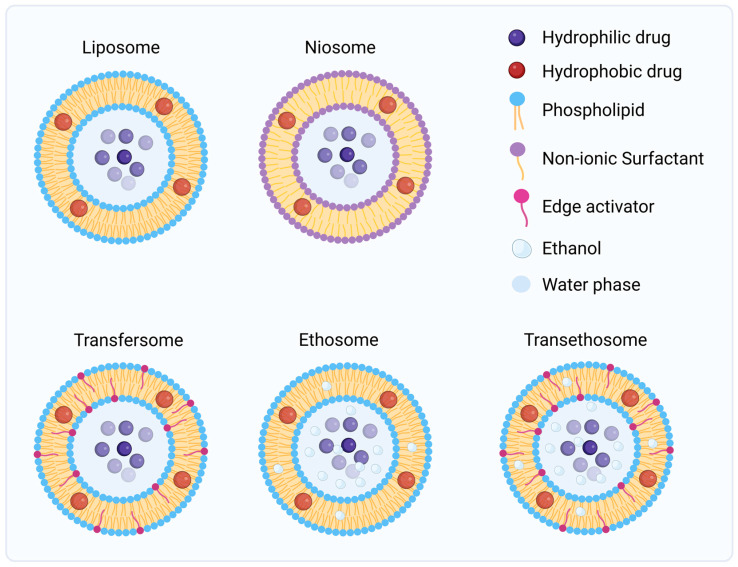
Schematic representation of lipid-based nanocarriers as topical delivery systems. *Created in BioRender. Secci, D. (2026) https://BioRender.com/iz7yzgg (Accessed on 1 December 2025)*.

In contrast, deformable vesicles, including transfersomes, ethosomes, and transethosomes, possess a more flexible membrane architecture designed to overcome the dense lipid packing of the skin barrier. Transfersomes incorporate edge activators such as Tween 80, Span 80, or sodium cholate that destabilize the bilayer, conferring high elasticity and enabling the vesicle to squeeze through intercellular spaces much smaller than its own diameter [83,85]. Ethosomes combine phospholipids with high ethanol concentrations, which fluidize both vesicle and skin lipids, facilitating deep penetration and enhanced dermal accumulation. Transethosomes, integrating both ethanol and an edge activator, exploit a synergistic mechanism that maximizes deformability, penetration depth, and drug retention. These flexible vesicles not only enhance the permeation of hydrophilic or macromolecular drugs but also improve cutaneous deposition by interacting dynamically with the lipid domains of the stratum corneum [77,78,84]. Figure 7 summarizes the structures of all the different types of lipid-based delivery systems.

The development of vesicular systems represents the natural evolution from rigid lipid assemblies to highly deformable nanocarriers specifically designed to traverse the complex lipid matrix of the stratum corneum. This progression has been particularly valuable in the context of skin cancer, where the therapeutic goal is to maximize local drug concentration within malignant tissue while minimizing systemic exposure [86,87]. By reproducing the amphiphilic structure of biological membranes, vesicular carriers combine biocompatibility and permeability enhancement, enabling efficient encapsulation and sustained release of both hydrophilic and lipophilic anticancer agents [79]. Their tunable composition allows optimization of particle size, surface charge, and deformability, which can be tailored to specific therapeutic goals such as localized drug retention in cutaneous tumors or controlled transdermal diffusion [77]. In skin cancer therapy, liposomes and related deformable vesicles have demonstrated enhanced penetration through the stratum corneum, improved drug deposition in the viable epidermis and dermis, and a consequent increase in therapeutic efficacy with reduced systemic exposure [86,87,88].

Despite these advantages, the clinical translation of these systems remains limited by challenges related to limited physicochemical stability, oxidative degradation of phospholipids, high production cost and manufacturing variability [78,84]. Nonetheless, their unique capacity to integrate structural adaptability and biological affinity places vesicular carriers among the most promising platforms for future topical and transdermal formulations in skin cancer management.

### 4.3. Hydrogels

Hydrogels are three-dimensional hydrophilic polymeric networks capable of absorbing and retaining large quantities of water or aqueous fluids while maintaining their structural integrity. Their porous, hydrated nature makes them particularly suited for local drug delivery, providing intimate contact with the skin surface and acting as a reservoir for sustained release [89].

Structurally, hydrogels can be prepared from natural polymers, such as chitosan, alginate, hyaluronic acid, gelatin, collagen, cellulose, or starch, which offer excellent biocompatibility and intrinsic bioactivity, or from synthetic polymers, including poly vinyl alcohol (PVA), PEG, poly acrylic acid (PAA), poly 2-hydroxyethyl methacrylate (PHEMA), PCL, and poloxamers [90]. In general, natural polymers offer biological affinity and a low toxicity profile, while synthetic ones provide superior mechanical strength, manufacturing reproducibility, and controlled degradation [91]. Hydrogels can also be formulated by combining natural and synthetic polymers, yielding hybrid networks that integrate the biocompatibility of natural components with the mechanical and physicochemical stability of synthetic materials [92].

The main advantages of hydrogels in skin delivery derive from their high water content and flexibility. They provide excellent skin compatibility and hydration of the stratum corneum, reducing transepidermal water loss and softening the barrier, which can facilitate drug permeation [93]. In topical formulations, cross-linking can occur through physical interactions, such as ionic or temperature-induced associations, or through chemical covalent bonding. These two different mechanisms define the mesh architecture and control the rate of drug release [94]. However, because standard hydrogels are mainly hydrophilic, their ability to incorporate lipophilic drugs is limited. To address this issue, amphiphilic copolymers or hydrophobic monomers are often introduced into the network to create localized hydrophobic domains that improve the loading of poorly water soluble compounds [95].

Despite these modifications, hydrogels alone exhibit limited penetration through the intact stratum corneum due to their hydrophilic nature and large molecular mesh size. Other challenges include formulation stability over time and the mechanical fragility of highly hydrated networks. As a result, they are frequently engineered as hybrid systems, incorporating nanoparticles, liposomes, or nanoemulsions within the gel matrix. These systems combine the occlusive and moisturizing effects of hydrogels with the permeation-enhancing properties of lipid or polymeric nanocarriers, thereby enhancing stability, drug loading and controlled release [96].

In the context of skin cancer therapy, hydrogels offer a non-invasive and localized drug delivery platform that can reduce systemic toxicity while maintaining therapeutic concentrations at the tumor site. Hybrid formulations have shown enhanced drug retention, sustained release, and improved tumor targeting compared with free drugs [97].

Overall, hydrogel-based systems contribute to prolonged local exposure, reduced systemic diffusion, and improved patient compliance, representing a complementary or synergistic platform alongside lipid and polymeric nanocarriers for advanced topical treatments.

### 4.4. Mechanical and Physical Enhancement Strategies

In addition to biomaterial-based nanocarriers, several strategies have been developed to overcome the barrier function of the stratum corneum. These techniques transiently disrupt or bypass the outer skin layer, enabling controlled delivery of therapeutic molecules to improve local bioavailability in skin cancer therapy.

Broadly, they can be distinguished into mechanical enhancement methods, which disrupt the skin to facilitate drug delivery, and physical enhancement methods, which rely on external stimuli such as heat, electric fields, or ultrasound to transiently increase skin permeability without causing permanent tissue disruption.

Microneedles are devices designed to pierce the stratum corneum and enable drug diffusion into the viable epidermis and dermis. They bridge the gap between conventional transdermal patches and hypodermic needles, providing a precise and tolerated route for localized or systemic drug administration [98].

Microneedles can be manufactured in various designs depending on the intended delivery mechanism and material composition. Solid microneedles, typically made of stainless steel, titanium, or silicon, are used to pierce the stratum corneum creating microchannels that enhance drug diffusion when a topical formulation is later applied. Coated microneedles are constructed from similar rigid materials but feature a thin drug layer deposited on their surface that dissolves rapidly upon insertion. Hollow microneedles, also produced from metals, silicon, or robust polymers, contain an internal cavity that enables the active infusion of liquid formulations under pressure. In contrast, dissolving microneedles are composed of biocompatible and water-soluble polymers such as hyaluronic acid, polyvinylpyrrolidone, polyvinyl alcohol, or dextran, which dissolve within the skin to release their encapsulated drug payload. Finally, hydrogel microneedles, often based on cross-linked polymers like gelatin methacryloyl (GelMA) or hyaluronic acid methacrylate (HAMA), swell upon contact with interstitial fluid, enabling sustained or responsive drug release [99,100].

Microneedles offer several advantages over topical formulations. Their minimally invasive nature allows precise dose localization and reduces systemic exposure. They are compatible with a wide range of therapeutic agents, including macromolecules, nanoparticles, and vaccines, and can be designed for controlled or rapid release depending on the material employed. However, limitations still exist including low drug-loading capacity, variability between different skin types, and manufacturing constraints related to scalability, cost and sterility [101]. Despite these limitations, this physical approach enhances local penetration while maintaining minimal invasiveness and patient comfort, making microneedles an increasingly attractive platform for the topical treatment of skin cancers and other dermatological conditions [102].

Physical enhancement methods employ external energy sources to increase skin permeability and improve the penetration of therapeutic agents. They can be broadly divided into thermal-assisted approaches, which use controlled heat or light to modulate skin structure and trigger drug release, and energy-driven techniques, which apply electrical or acoustic stimuli to create reversible pathways across the stratum corneum.

Thermal-based approaches involve the controlled application of heat to fluidize stratum corneum lipids and accelerate diffusion or trigger drug release from thermoresponsive systems such as liposomes and polymeric hydrogels [103]. Photothermal therapy (PTT) uses light-absorbing nanomaterials, commonly gold nanorods, graphene oxide, or polydopamine nanoparticles to convert near-infrared radiation into localized heat, enabling selective tumor ablation. When combined with drug-loaded nanocarriers, chemo-photothermal synergy can be achieved [104].

Other non-invasive approaches include iontophoresis, that enhances drug permeation by applying a low-intensity electric current across the skin via conductive electrodes. The electric field promotes the movement of charged and polar molecules via electrophoresis and electro-osmosis, facilitating their controlled transport through the stratum corneum and into deeper tissues [105].

Electroporation, instead, employs short high-voltage electrical pulses to create transient aqueous pores within the lipid domains of the stratum corneum. This temporary permeabilization increases skin conductivity and facilitates the passage of both small molecules and macromolecules [106]. Lastly, sonophoresis utilizes low-frequency ultrasound waves to enhance skin permeability by inducing acoustic cavitation and microstreaming within the stratum corneum. The disruption of the lipid organization increases the fluidity of intercellular domains and promotes the diffusion of both hydrophilic and lipophilic drugs across the skin barrier [107].

Collectively, these methods offer controllable and reversible enhancement of transdermal transport while preserving tissue integrity. They have been increasingly explored in recent years for skin cancer therapy, particularly in combination with conventional topical and/or nanocarrier formulations [13,16].

## 5. Current and Future Perspectives in Topical Treatment of Skin Cancers

Topical therapy is an established method for treatment of skin cancers, with many variable factors to take into consideration to achieve good delivery. Its clinical efficacy depends predominantly on the biology of the malignant cell, the depth of tumor invasion into the epidermis and superficial dermis [43]. Non-melanoma skin cancers (NMSC) are particularly susceptible to topical treatment because their most common subtypes, actinic keratoses, squamous cell carcinoma in situ, and superficial basal cell carcinoma, remain confined to epidermal structures, allowing drug penetration to reach the full extent of the lesion. In contrast, melanoma typically becomes invasive at an early stage and rapidly gains access to the dermis, placing the malignant cells beyond the reach of conventional topical agents [5,7]. This biological difference explains why several topical drugs are used in NMSC treatment, whereas their role in melanoma is still extremely limited.

Melanoma represents the most challenging skin cancer to treat, and topical therapy is ineffective in most cases because invasive melanoma swiftly breaches the basement membrane and disseminates through dermal lymphatic channels. As a result, topical treatment is limited to two clinical cases: melanoma in situ (MIS), and selected cases of superficial in-transit metastases [10,27].

In MIS, malignant melanocytes remain confined to the epidermis, making them accessible to topical immunomodulators. 5% imiquimod cream is the only topical agent consistently used in treatment of MIS, especially for lesions located on cosmetically sensitive facial areas or for patients who are not surgical candidates [108]. Its mechanism of action relies on TLR7-mediated activation of local immune responses, promoting cytotoxic T-cell infiltration and clearance of atypical melanocytes [13]. A second, more specialized indication involves small, superficial in-transit metastases, which consist of metastatic deposits arising within dermal or subcutaneous lymphatic vessels. In these cases, imiquimod may be applied after debulking methods such as shave excision, curettage or laser ablation to eliminate residual tumor nests [27,109].

By contrast, topical therapy is an integral and well-validated treatment for several forms of NMSC. Superficial basal cell carcinoma (sBCC), due to its confinement to the epidermis and its minimal risk of metastasis, responds particularly well to topical approaches [110]. 5% imiquimod cream is widely used in sBCC treatment, providing durable responses through local immune stimulation, while 5-fluorouracil (5-FU) 5% cream offers an effective cytotoxic alternative by inhibiting thymidylate synthase in rapidly proliferating keratinocytes [111]. Photodynamic therapy (PDT) represents another cornerstone of topical NMSC treatment. Following application of 5-aminolevulinic acid (ALA) or methyl-aminolevulinate (MAL), tumor cells accumulate the photosensitizer protoporphyrin IX, which upon illumination with red light generates reactive oxygen species that selectively destroy malignant tissue [112,113].

A similarly favorable therapeutic window exists for squamous cell carcinoma in situ, in which atypical keratinocytes remain restricted to the epidermis. Both imiquimod and 5-FU achieved high clearance rates, and PDT is frequently employed for the treatment of this disease [114,115]. Actinic keratoses (AK), the precursor lesions to squamous cell carcinoma, also respond well to topical therapies with 5-FU, imiquimod, diclofenac gel and ALA/MAL-PDT widely used in clinical practice. However, once squamous cell carcinoma becomes invasive and extends into the dermis, topical agents are no longer effective, and surgical management is mandatory [116,117,118].

Although current clinical practice is dominated by conventional cream formulations and photodynamic therapy, the progress made in the design of biomaterial-based delivery systems has significantly expanded the therapeutic opportunities for topical and transdermal skin cancer treatment [13,48]. Across polymeric nanoparticles, lipid-based formulations, hydrogels, and physical enhancement technologies, several strategies have demonstrated clear potential to improve dermal penetration, enhance local drug accumulation, and reduce systemic exposure. A common finding among these platforms is their ability to stabilize otherwise labile anticancer compounds, increase their residence time within the epidermis and dermis, and promote controlled delivery directly to malignant tissue [43,48,87].

Polymeric nanoparticles have demonstrated considerable potential for topical skin cancer therapy. Polymeric nanocapsules loaded with imiquimod or 5-fluorouracil and incorporated into bioadhesive gel formulations have been shown to enhance drug penetration and retention within epidermal and dermal layers, while improving in vitro cytotoxicity against melanoma and basal cell carcinoma models [119,120].

Similarly, paclitaxel-loaded polymeric micelles embedded in Carbopol hydrogels enhanced cutaneous delivery, resulting in increased skin retention and significant tumor growth inhibition in melanoma-bearing mice following topical administration [121].

More advanced translational evidence has been provided using human-derived 3D models, where topical application of doxorubicin-loaded PLGA nanoparticles reduced tumor thickness, suppressed proliferation, and induced apoptosis in both melanoma and cutaneous SCC [122].

Collectively, these studies highlight the versatility of polymeric nanocarriers in facilitating localized drug delivery and support their continued development as complementary platforms for topical skin cancer treatment.

Among the different approaches, lipid-based nanocarriers remain promising strategies. Nanoemulsions have shown enhanced delivery of agents such as 5-fluorouracil, dacarbazine, and curcumin, achieving greater dermal penetration and retention than conventional formulations [123,124,125]. Their small droplet size increases contact with the stratum corneum, while surfactant-induced lipid fluidization promotes diffusion across the typically rigid and hyperkeratotic barrier of tumor-affected skin, enabling more efficient access to deeper epidermal layers [13,64].

SLNs and NLCs have improved dermal deposition of various chemotherapeutics such as 5-FU, doxorubicin, resveratrol and paclitaxel by forming occlusive films, promoting lipid exchange with the stratum corneum, and providing sustained drug release [126,127,128,129]. Their biocompatible composition and the ability to incorporate drugs within crystalline or imperfect lipid matrices make these carriers particularly suitable for chronic or repeated topical application in non-melanoma skin cancers.

Vesicular systems have also shown strong potential, particularly for agents that are difficult to deliver across the skin barrier. Ethosomes, transfersomes, and other deformable vesicles have demonstrated improved penetration and localized retention of compounds such as methotrexate, imiquimod, doxorubicin, and resveratrol, owing to their flexible bilayers and ability to fluidize stratum corneum lipids [130,131,132,133]. Transferosomes have shown notable depth of penetration in preclinical models due to their high deformability, and are increasingly being explored for combination therapy, including the co-delivery of chemotherapeutic agents with photosensitizers [88].

Hydrogel-based systems represent another emerging direction, especially when used as hybrid platforms incorporating nanocarriers within the hydrogel matrix. These formulations have shown improved local delivery of 5-FU, curcumin, leflunomide and other plant-derived anticancer agents by combining the hydrating, barrier-softening properties of hydrogels with the enhanced penetration and controlled release of nanoparticles and vesicles [97,134,135]. Their ability to conform to irregular lesion surfaces and maintain prolonged contact with the skin makes them particularly attractive for the management of superficial basal cell carcinoma, melanoma in situ, and actinic keratoses [96].

Mechanical and physical enhancement techniques are also advancing toward more effective and patient-compatible solutions. Microneedles have shown great promise for delivering both small molecules and complex nanocarriers directly into the viable epidermis and dermis, improving the effectiveness of drugs such as doxorubicin, cisplatin, and 5-FU in preclinical settings [136,137,138,139,140]. Their ability to create microchannels through the stratum corneum enables controlled release while maintaining minimal invasiveness [141]. Physical methods such as iontophoresis, electroporation, and sonophoresis have further demonstrated enhanced permeation of chemotherapeutics and immunomodulatory agents, supporting their use in combination with optimized biomaterial-based formulations [142,143,144,145].

Among the biomaterials discussed, several polymeric materials stand out due to their established clinical use and translational relevance across biomedical applications [146]. PLGA, for example, is an FDA-approved synthetic biodegradable polymer widely employed in long-acting injectable depots, implantable drug delivery systems, and microsphere-based formulations for endocrine, oncological, and neurological indications, providing an established regulatory and safety profile that supports its continued exploration as a nanocarrier material for topical skin cancer therapy [147].

Similarly, polymeric hydrogels based on clinically established materials such as hyaluronic acid, alginate, and PEG have achieved broad clinical use in dermatology and regenerative medicine, particularly as wound dressings, injectable depots, and topical delivery matrices [148]. Their biocompatibility, high water content, and tunable mechanical properties enable prolonged contact with the skin and support localized drug delivery, making them attractive platforms for the development of hybrid delivery systems for topical oncological applications.

Looking forward, the most promising advancements are likely to come from integrated systems that combine multiple permeation-enhancing mechanisms within the same platform. Formulations that pair nanocarriers with microneedles, or nanocarriers within hydrogel matrices, provide complementary advantages in terms of penetration depth, controlled release, and patient comfort. The development of stimuli-responsive materials, such as pH-sensitive, enzyme-responsive or thermally activated systems may enhance the control of drug release in the tumor microenvironment and limit systemic exposure.

Overall, the convergence of biomaterial innovation, improved physicochemical understanding of the skin barrier, and more advanced in vitro and ex vivo models is expected to accelerate the translation of these technologies into clinical practice. While challenges related to stability, manufacturing, scale-up, and regulatory classification remain unsolved, the results achieved with nanoemulsions, polymeric and lipid nanoparticles, vesicular systems, hydrogels, and microneedles illustrate a clear path toward more effective and localized topical therapies. The continued refinement of these platforms, particularly those demonstrating improved penetration and controlled release of clinically relevant anticancer agents, holds strong potential to expand non-invasive treatment options for both melanoma and non-melanoma skin cancers.

## Data Availability

No new data were created or analyzed in this study. Data sharing is not applicable to this article.

## Abbreviations

The following abbreviations are used in this manuscript:

5-FU	5-fluorouracil
AK	Actinic keratoses
ALA	5-aminolevulinic acid
BCC	Basal cell Carcinoma
GelMA	Gelatin methacryloyl
HAMA	Hyaluronic acid methacrylate
MAL	Methyl-aminolevulinate
MIS	Melanoma in situ
NLCs	Nanostructured lipid carriers
NMSC	Non-melanoma skin cancers
PAA	Poly acrylic acid
PCL	Poly ε-caprolactone
PDT	Photodynamic therapy
PEG	Polyethylene glycol
PHEMA	Poly 2-hydroxyethyl methacrylate
PLA	Poly lactic acid
PLGA	Poly lactic-co-glycolic acid
PTT	Photothermal therapy
PVA	Poly vinyl alcohol
sBCC	Superficial basal cell carcinoma
SCC	Squamous cell carcinoma
SLNs	Solid lipid nanoparticles
